# Review of Studies Concerning Electromagnetic Field (EMF) Exposure Assessment in Europe: Low Frequency Fields (50 Hz–100 kHz)

**DOI:** 10.3390/ijerph13090875

**Published:** 2016-09-01

**Authors:** Peter Gajšek, Paolo Ravazzani, James Grellier, Theodoros Samaras, József Bakos, György Thuróczy

**Affiliations:** 1 Institute of Non-Ionizing Radiation (INIS), Pohorskega Bataljona 215, Ljubljana 1000, Slovenia; 2 Istituto di Elettronica e di Ingegneria dell’Informazione e delle Telecomunicazioni IEIIT, CNR Consiglio Nazionale delle Ricerche, Piazza Leonardo da Vinci 32, Milan 20133, Italy; paolo.ravazzani@ieiit.cnr.it; 3 European Centre for Environment and Human Health (ECEHH), University of Exeter Medical School, Knowledge Spa, Royal Cornwall Hospital, Truro, Cornwall TR1 3HD, UK; J.Grellier@exeter.ac.uk; 4 Formerly Centre for Research in Environmental Epidemiology (CREAL), Parc de Recerca Biomèdica de Barcelona, Doctor Aiguader, 88, Barcelona 08003, Spain; 5 Department of Physics, Aristotle University of Thessaloniki, Thessaloniki GR-54124, Greece; theosama@auth.gr; 6 National Research Institute for Radiobiology and Radiohygiene, Anna u.5., Budapest 1221, Hungary; bakos.jozsef@osski.hu (J.B.); thuroczy.gyorgy@osski.hu (G.T.)

**Keywords:** electromagnetic fields, exposure assessment, exposimetry, ELF magnetic fields, intermediate frequencies (IF)

## Abstract

We aimed to review the findings of exposure assessment studies done in European countries on the exposure of the general public to low frequency electric and magnetic fields (EMFs) of various frequencies. The study shows that outdoor average extremely low frequency magnetic fields (ELF-MF) in public areas in urban environments range between 0.05 and 0.2 µT in terms of flux densities, but stronger values (of the order of a few µT) may occur directly beneath high-voltage power lines, at the walls of transformer buildings, and at the boundary fences of substations. In the indoor environment, high values have been measured close to several domestic appliances (up to the mT range), some of which are held close to the body, e.g., hair dryers, electric shavers. Common sources of exposure to intermediate frequencies (IF) include induction cookers, compact fluorescent lamps, inductive charging systems for electric cars and security or anti-theft devices. No systematic measurement surveys or personal exposimetry data for the IF range have been carried out and only a few reports on measurements of EMFs around such devices are mentioned. According to the available European exposure assessment studies, three population exposure categories were classified by the authors regarding the possible future risk analysis. This classification should be considered a crucial advancement for exposure assessment, which is a mandatory step in any future health risk assessment of EMFs exposure.

## 1. Introduction

The general public is exposed to an ever-increasing number of sources of electromagnetic fields from various electric devices, appliances and technologies (i.e., transmission lines, transformers, typical household appliances and their power supplies). Time-varying electric and magnetic fields with a frequency of less than 10 MHz are defined in this paper as being low frequency (LF). In the scientific literature, this frequency band is split into two main ranges, namely extremely low frequency (ELF) with frequencies up to 300 Hz, and intermediate frequency (IF) with frequencies between 300 Hz and 10 MHz. 

The most prominent frequency in Europe is 50 Hz (often called mains, utility or power frequency) and its harmonics, particularly the third one. Electric trains are operated at about 16.7 Hz in some European countries, so public exposures at this frequency have also been taken into account [1]. The main sources of exposure for the general public are household electric appliances, and billions of these devices are used in everyday life in Europe. The related exposures are localized and their magnitude strongly depend on the distance of the user from the appliance itself [2].

The outdoor sources of ELF-EMFs exposure with the greatest spatial coverage are high-voltage overhead power lines, which are used for the transmission and distribution of electricity. There are approximately 360,000 km of overhead transmission power lines in Europe, operating at voltages of between 110 and 400 kV [3,4]. Underground cables are also used to deliver electric power. Approximately 2% of all electric power in Europe is supplied by underground cables [3]. Such cables can produce magnetic fields directly above them (i.e., along the line of the route itself) stronger than those associated with aerial lines, due to the smaller distance between the external environment and the cable itself (typically a few meters). For instance, 400 kV underground cables can produce a magnetic field intensity of over 30 µT at ground level, falling to 10 µT at 2 m above the ground, since the field intensity falls very rapidly with increasing distance from either side. Careful arrangement of the conductors and phase optimization may be done so that the spatial extension of the magnetic field is minimised [5]. 

Various automobile components require electric energy. These produce low-frequency magnetic fields in the cables and components that conduct the electricity. The frequency range of such fields is wide, ranging from a few Hz to several kHz. Hybrid cars produce stronger magnetic fields than traditional vehicles fitted with only petrol or diesel engines [1,6]. 

Sources of IF fields include computer and television screens containing cathode ray tubes, anti-theft devices in shops [7], toys including electric engines, and induction cookers/hotplates. The reference levels for exposure of the general public might be exceeded in the immediate vicinity of such devices. Some other sources of IF have recently become extremely common in homes, such as compact fluorescent lamps (energy-saving light bulbs), and other devices may soon follow suit, for example appliances for wireless inductive charging of batteries [8].

In this panorama, the International Commission on Non-Ionizing Radiation Protection (ICNIRP) published guidelines suggesting limits of exposure to ELF-EMFs in 1998 [9], updating them in 2010 [10]. In this paper, “exposure limits” will always refer to the ICNIRP limits.

In this review, we aimed to present a survey of the data available for European countries relating to the exposure of the general public to low frequency EMF of various frequencies and sources. We present the results in terms of the various measurement approaches used in Europe to measure fields related to ELF-EMFs sources, discussing the available measurement data that we have identified.

## 2. Exposure Assessment and Monitoring in Europe

Efforts have been made to determine the exposure of the public to EMFs of various frequencies in several European countries. Different methods of exposure assessments have been used in Europe over the last three decades including: (i) in-situ measurements (indoor and outdoor spot measurements; measurements of magnetic fields around electric devices and household electric appliances); (ii) personal (or individual) exposimetry; (iii) modelling of exposures on the basis of the configuration of wiring systems. These activities are summarized in Table 1.

### 2.1. Spot Measurements in Outdoor and Indoor Environments

Many measurements have been carried out worldwide around high-voltage power lines in order to map the spatial distribution of ELF-EMFs. According to these studies, a large electricity pylon carrying a 380/400 kV conductor produces a magnetic field of around 10–20 μT directly under the pylon itself, and an electric field of around 3–5 kV/m. These levels fall with distance (inverse square law) from the sides of the line [5]. 

In this paper, we are interested in describing the exposure of the general public to ELF-EMFs, rather than those in close proximity to power lines alone, for example in urban, rural, indoor, outdoor environments across several European countries. Exposure measurements of ELF-MF in urban environments have been performed in Spain, Italy, Sweden, Norway and the UK. In Spain, spot measurements were made in five cities. The average magnetic field was 0.2 µT, ranging from 0 to 7 µT [11,12]. In Sweden, ELF-MF along certain stretches of sidewalk in the centre of Goteborg were mapped. About 50% of the investigated streets had magnetic fields of the same order of magnitude. The median value of magnetic field flux density was 0.2 µT [13]. Similar surveys were conducted in Turin, Italy, where measurements were made while walking at normal speed along an established path. The measured data, consisting of more than 100,000 samples, did not follow a normal distribution, and showed an arithmetic mean (AM) of 0.19 µT, a median of 0.08 µT and a geometric mean (GM) of 0.06 µT [14]. Between 2006 and 2008, outdoor and indoor measurement campaigns were carried out in the town of Aosta in Italy. The highest outdoor measurement of 80 µT was measured against the wall of a transformer box; this decreased to 1.34 µT at 1 m from the wall [15]. In order to evaluate seasonal variations due to changes in power consumption, ELF magnetic fields were measured in summer and winter in Norway. Magnetic fields were mapped at 1.0 m above the ground. In summer, less than 4% of the streets had values exceeding 0.4 µT, but this increased to 29% and 34% on cold and on snowy winter days, respectively. The average levels were 0.13 µT (summer), 0.85 µT (winter, cold), and 0.90 µT (winter, snow), with the highest recorded value of 37 µT [16]. The exposure of the general public to Belgian power distribution substations that transform the voltage from 11 to 0.22–0.4 kV were reported by Joseph et al. [17], with magnetic field values within the range of 0.025–47.4 µT. Average exposures of 0.4 µT were obtained at a minimum distance of between 5.4 m (average day) and 7.2 m (average year). In the UK, the NRPB (now part of Public Health England) has performed measurements on 27 substations. Typical values at the perimeter fence were 10 μT for 275 and 400 kV substations, and 1.6 μT for an 11 kV substation. The mean field at the substation boundary was 1.1 μT, with a field of 0.2 μT up to 1.5 m from the boundary [18]. 

In summary, the outdoor average ELF-MF in public areas in urban environments ranges from 0.05 to 0.2 µT, but stronger values may occur directly beneath high-voltage power lines, at the wall of transformer buildings, and at the boundary fences of substations. In the case of the latter, the maximum field can reach up to 20–80 µT. 

#### 2.1.1. Residential Exposure

For residential exposure, the major sources of magnetic fields are household appliances, nearby power transformers and high-voltage transmission lines, and domestic appliances. The largest measurement study of residential exposure assessment was performed in Germany with measurements of magnetic field at 50 Hz and 16.7 Hz made in 1835 fixed-location residences [19]. Fields were measured in children’s bedrooms for 24 h. Median 50 Hz magnetic fields above 0.2 µT were found only in 1.4% of homes. Stronger magnetic fields were less frequently encountered: 0.3 μT was found in 0.4%, and 0.4 μT was found in 0.2% of residences. The median magnetic field produced by high-voltage power lines (123–420 kV) was above 0.2 µT in only 8 of 25 residences (32.0%) that were located 50 m or closer to a power line. A clear association was found between residential magnetic field intensity and the type of the residence, with stronger average fields in apartment buildings.

In the UK, a week-long survey of power-frequency magnetic field measurements was conducted in 258 homes [1]. The factor that was found to most strongly influence exposure in the home was the presence or absence of overhead power lines at voltages of ≥132 kV within 100 m from the home. The geometric-mean of time-weighted average fields measured was of about 0.2 µT within 100 m from the lines, and 0.054 µT at distances greater than 100 m.

Similar studies were carried out in Austria in 2006 with spot measurements made at the bedside in 226 households [20]. Average night-time ELF-MF above 0.1 µT were obtained in 2.3% of households. Higher ELF electric fields were primarily associated with presence of lamps beside the bed (maximum of 166 V/m). Higher ELF-MF were attributed to the transformers of these devices (maximum of 1.03 µT) or high current in the power supplies (maximum of 0.38 µT). The average night time arithmetic mean of the ELF-MF was above 0.1 µT. The highest ELF electric fields were primarily due to lamps next to the bed, with a maximum of 166 V/m; the highest ELF-MF were due to transformers of devices, with a maximum of 1.03 µT or high current of power lines, with a maximum of 0.380 µT [20]. In 2009, Tomitsch and Dechant repeated the same measurements of the electric and magnetic fields radiated by the same households surveyed in 2006 [21]. The results showed that the median electric field levels had decreased to 17.35 V/m. The median magnetic fields had decreased to 12.76 nT, whereas the arithmetic mean remained almost unchanged. Simple measures taken to reduce exposure resulted in an average decrease to 0.023 µT for the magnetic fields and to 23 V/m for the electric fields. Typical reduction measures employed were the removal or rearrangement of clock radios and transformers of devices, removal or rearrangement of bedside lamps, rearrangement of extension cables or socket adaptors, removal of fuses or change of phase and neutral line [20]. 

In the UK, both electric and magnetic fields were measured in 549 homes within the United Kingdom Childhood Cancer Study [22]. In a follow-up study, 196 homes were identified and categorized into high (>0.2 µT) and low (<0.2 µT) exposure [18]. Possible sources of these exposures were also analysed. In 102 homes with fields estimated at or above 0.2 µT, exposure was attributed to low voltage (0.4 kV) supplies to the home. 

In Sweden, ELF field measurements were performed in 100 houses, randomly selected to be in either urban areas or in the countryside [23]. The study found that almost 90% of the measured houses had magnetic fields below 0.2 μT, with a mean value of 0.11 μT and a median value of 0.05 μT. The magnetic field was found to be highest on the ground-floor as compared to the middle or top floors. This was due to underground heating systems and the electric wiring in the houses. Total harmonic distortion was found to be high in some houses and the reason for this was the large amount of non-linear loads. 

In Italy, a survey was performed in different rooms of 37 houses with no nearby overhead power lines [24]. The field levels were measured under “power-off” (i.e., household devices were turned off) and “power-on” (i.e., household devices were turned on) conditions. The average magnetic field level was stronger in apartments (0.8 μT) than in houses (0.3 μT). 

Data from various countries show that the geometric mean of spot measurements in homes do not vary strongly. In Finland, the geometric mean is 0.060 µT, between 0.026 and 0.029 µT in Germany, between 0.037 and 0.048 µT in Sweden, and 0.029 and 0.064 µT in the UK. These data should, however, be interpreted with caution, given great differences in the evaluation conditions [1]. 

A survey commissioned by the Swiss Federal Office of Public Health set out to measure the magnetic fields produced by various electric floor heating systems. The largest mean magnetic fields (up to 1.3 µT taken in a grid of 20 cm squares, 50 cm above floor level) were produced by the systems with single-core heating cables. Though the levels were found many times below the international guidelines, they far exceeded the magnetic fields normally encountered in dwellings [25].

#### 2.1.2. Measurement Close to Transformer Stations

In some European countries, transformer stations (from 0.4 to 10 kV) are installed in multilevel residential buildings [26]. These built-in transformer stations may elevate the magnetic field exposure in the rooms directly above the station. The source of these magnetic fields is mainly the distribution bars (so-called “bus bars”) that are typically mounted on the wall and/or ceiling of the room containing the transformer. These magnetic fields have been observed to reach some tens of μT on the floor above the transformer room, but decreased to a few μT at a distance of 1 m above the floor. These results are consistent with a study performed in Hungary, where mean magnetic field exposures of a number of rooms of apartments were characterized using five spot measurements made at a height of 1 m above the floor [27]. The results of the measurements in 31 multilevel buildings showed that in the apartments just above the transformer station the mean exposure to 50 Hz magnetic fields was 0.98 µT, whereas in the apartments on other upper floors it was only 0.1 µT. In another study, time-weighted average personal exposures in apartments above the transformer were found to be 0.825 and 1.033 µT, for home and in bed, respectively [28]. 

In a Finnish study, the mean of spot measurements was found to be 0.62 µT in the apartments immediately above transformers, 0.21 µT for apartments on the first floor, and 0.11 µT in apartments on other upper floors [29]. Similar studies have been conducted in Switzerland where the magnetic field was 0.59 μT on average in apartments that were either directly above the transformer or were in contact with the transformer room, but exposure was reduced to 0.07 μT in apartments which did not touch any wall of the transformer buildings [30]. Preliminary results from Bulgaria show that in apartments above the transformers the average magnetic field was 0.4 μT, while on other floors it was 0.1 μT [26]. In 2011, Röösli and colleagues measured the ELF-MF levels in 39 apartments in 18 buildings. Measured arithmetic mean ELF-MF was 0.59 μT in eight apartments that were fully adjacent to a transformer room. In apartments that only partly touched the transformer room at corners or edges, average ELF-MF level was 0.14 μT [30]. Average exposure in the remaining apartments was 0.10 μT. They found a distinct ELF-MF exposure gradient in buildings with transformers and concluded that exposure classification based on the location of the apartment relative to the transformer room appears feasible. 

Yitzhak and colleagues performed a 24-h measurement of 50 Hz magnetic fields in apartments of buildings containing transformer stations. They found that average magnetic field in apartments above the transformer room were 0.33 µT, significantly stronger than those measured in apartments positioned at greater distances from the transformer station (from 0.06 to 0.11 µT) [31].

#### 2.1.3. Measurement of Exposure Due to Transport Systems

Relatively few studies have been conducted on ELF exposure levels in and around transport systems such as trains, trams and hybrid cars. The maximum levels of recorded magnetic field strength are emitted at 50 Hz in a tram, at 15.25–16.50 Hz in a train, and at 12 Hz in a hybrid car [6,32]. According to World Health Organization (WHO), peak magnetic fields of up to a few tens of μT have been recorded on the platform of a local city railway line [1]. In France, measurements performed in 1993 inside a high-speed train, and at a distance of 10 m outside the train, showed peak values around 6–7 μT during high-speed drive [33], although these data are quite dated, considering the technological changes and evolution of the last 25 years. In a Swedish study [34], field values in the driver cabin ranged from a few μT up to over 100 μT, with mean values for a working day between a few μT up to tens of μT depending on the engine. The measurements of magnetic field strength in the front of a train at floor level were in the range of 3.4–8.7 µT. In a tram, the peak magnetic field strength of 7.6 µT was recorded in the middle of the tram on the floor level when another tram passed in close proximity. The magnetic field strength near the floor on the outside of the tram reached up to 3.5 µT when a tram passed on the rail. Most of the field strength was in the range of 0.01–5.5 µT. The range of magnetic fields in a hybrid car was found to be 0.03–2.4 μT. Low frequency magnetic fields (5 Hz–2 kHz) in all four seats of seven stationary cars were determined with their engine and air conditioning running. Magnetic fields averaged over the body ranged from 0.03 to 4 μT. At the left rear seat, a maximum magnetic field of 14 µT was measured at foot level. The Swiss Federal Office of Public Health (FOPH) commissioned a study in which the magnetic fields produced by car tyres were measured. Since the low-frequency magnetic fields are produced when the magnetic tyres rotate, measurements were made in cars travelling at 80 km/h. The magnetic fields were measured at frequencies from 5 Hz to 2 kHz in 12 different cars. Higher values were measured in the foot area of the passenger seat and on the back seat. In 33% of the cars, values above 2 µT were measured; in 25% of the cars values were above 6 µT. The fundamental frequency of the magnetic fields is 10–12 Hz at a speed of 80 km/h. However, stronger harmonic frequencies were also measured [35,36]. 

More recently, Tell and colleagues [6] conducted a pilot study to assess magnetic field levels in electric compared to gasoline powered vehicles. They found that the geometric mean of all measurements was 0.095 µT in seven electric cars, compared to 0.051 µT in four gasoline-powered cars. Moreover, they compared the measured magnetic field level with the data from a previous exposure assessment of residential exposure in eight geographic regions in the United States. The results showed that the broadband magnetic fields in electric vehicles covered the same range as personal exposure levels recorded in that study.

#### 2.1.4. Exposure to Intermediate Frequencies (IF)

Only a few studies have dealt with exposure of the general public to IF fields (i.e., electronic article surveillance, energy saving bulbs, induction cookers). Electronic article surveillance (EAS) systems are used in many applications to prevent theft in boutiques, shops, supermarkets and libraries. The results of the study showed that the reference levels for most spatial locations of the grid were exceeded [7]. Bakos et al. showed that the maximum electric field strength in the 1.2–100 kHz frequency range in close proximity to the lamps was >42 V/m. In nine cases, the field strength exceeded 87 V/m and the highest measured value was 216 V/m [37]. The current densities in the tissue in the immediate vicinity of the lamp could reach between 10% and 55% of the exposure limit. The currents weaken rapidly with increasing distance from the lamps, and are only between 2% and 10% of the exposure limit at a distance of 20 cm. By contrast, the low- and medium-frequency magnetic fields are small and do not cause any significant exposure. Kos et al. reported that magnetic fields produced by induction cookers do not cause the basic restriction for the internal electric field according to the ICNRIP reference levels of 2010 to be exceeded in adults, children and foetuses, even when the fields are increased by a factor of 5 [10,38]. 

### 2.2. Personal Exposimetry

Data collected from spot measurements in outdoor and indoor environments do not provide sufficient insight into the exposure levels of individuals, who are often exposed to multiple sources at the same time and who are frequently moving through a variety of environments during a typical day. Capturing the true EMF exposure experienced by an individual requires that explicit measurements be made across time and space, using, for example, personal exposimeters.

Monitoring the individual magnetic field exposure of a subject using personal exposimeters (also called “dosimeters”) that are worn on the body is an attractive proposition because it allows the recording of exposure to fields from all sources and in any environment in which the individual is located. Since for all practical purposes the human body does not perturb the ELF-MF, the recorded magnetic field may represent a reliable estimation of the real exposure levels. The values of magnetic field exposure recorded by personal (isotropic) meters tend to be stronger than those derived from spot or long-term measurements in homes, because the device will measure the field from all sources [1]. Most of the studies based on personal exposimeter measurements studies have been performed using various different models of the same type of device (EMDEX, EnerTech, Campbell, CA, USA) both in Europe and elsewhere. 

Personal ELF-MF exposimetry studies have been performed in a number of European countries in the last decades, including Italy, Germany, France, United Kingdom, Denmark, Switzerland, Slovenia, Austria, and Hungary. The largest studies were conducted in Germany, France, and Switzerland. 

In the framework of the FP7 European Project ARIMMORA, personal measurement campaigns have been implemented in Italy and Switzerland, as a means of better describing the everyday exposure of children to ELF-MF [39]. In the two countries, 172 children aged between 5 and 13 years were equipped for 48 h with personal measurement devices (EMDEX II) to record ELF-MF exposure during their regular activity. These measurements have been performed twice, in both summer and winter. In addition, 24 h measurements were taken in the bedroom of the children. In order to maximize the exposure situations children living or attending school within 200 m of a high voltage power line (HVPL; ≥132 kV) or within 50 m of an underground cable and children living in a building with built-in transformer station were oversampled. The average geometric mean for personal ELF-MF exposure was 0.04 µT and for exposure in bedrooms was 0.05 µT. Living or attending school within 100 m of a voltage power line higher than 132 kV increased mean exposure by a factor of 3.5 and bedroom measurements by a factor 6.9 compared to a control group exposed to neither power lines nor in-building step-down transformers. The companion study limited to the ELF-MF exposure of children in the city of Milan in Italy [40] has shown a maximum magnetic field level in terms of average geometric mean ranging from 20 to 80 nT, well below the value of 0.3–0.4 µT applied in epidemiological studies for clustering the collected data about magnetic field levels. 

In Germany, individual magnetic field measurements at 50 Hz and 16.7 Hz were performed between 1996 and 1997 in 1952 people selected from the Bavarian population. The average of the individual flux density means for all participants amounted to about 0.1 µT and that of the individual medians was 0.047 µT at 50 Hz. Only 2.4% of the subjects had individual medians stronger than 0.2 µT. In only 31 measurements, the magnetic field exceeded 100 µT at 50 Hz, for a total time of only about 21 min, which was less than 0.001% of the total time for all measurements (5.3 years). For persons living next to railway lines, the mean individual magnetic field at 16.7 Hz was found to be 0.156 µT, and the mean individual median was 0.102 µT [41].

In Switzerland, a study with 552 volunteers was performed over a 24-h period in order to assess typical levels of 50 Hz magnetic fields and to identify the main causes for elevated field levels. The participants were selected from different professions and areas of northern Switzerland. The daily averages were found to be below 0.2 µT for 75% of the volunteers and in only the 3% of the cases this value was found above 1 µT. The measurement data had approximately lognormal distribution [42]. 

In Denmark, electric and magnetic fields were measured with personal dosimeters for 24 h, and the mean values were calculated for the measured levels in both working and non-working periods. The magnetic field exposure in residences away from power lines was 0.04 µT, and in residences near power lines it was 0.29 µT [43]. According to a recent study in Austria, the median exposure levels both at ELF and radiofrequency fields in residential areas were measured in 226 households. The magnetic and the electric field of the night-time ELF electric and magnetic field exposure close to the bed was found to be less than 0.05 μT and 26.2 V/m, respectively [20]. 

The feasibility of measuring exposure to ELF-MF in the UK Adult Brain Tumour Study was examined in 81 individuals in the United Kingdom. Exposure data were collected for 3–4 consecutive days. Data were collected over a total of 321 days, including non-occupational periods. There were no statistically significant differences between the populations in exposure during travel and domestic periods. The average daily exposure was 0.13 µT while exposure for domestic periods was 0.08 µT. The results also demonstrated that occupational exposure was the main determinant of overall exposure [44]. 

In 2006, the French government initiated a large study of personal exposure of the French population to 50 Hz magnetic fields [45]. The objective was to collect a database of 1000 children (0–14 years) and 1000 adults. The exposure data were collected during three measurement campaigns between 2007 and 2009. The arithmetic mean (AM) and geometric mean (GM) were 0.09 μT and 0.02 μT for children, and 0.14 μT and 0.03 μT for adults, respectively. Exposures for in the awake periods were 0.05 μT (AM) and 0.02 μT (GM) for children, and 0.10 μT and 0.03 μT for adults. The percentage of children with a 24 h mean exposure stronger than 0.4 μT was 3.1% (AM), and 0.2% (GM). The highest exposures for adults have been found in 11 cases, with a 24 h mean exposure stronger than 1.54 μT (AM), and 0.26 μT (GM). It was found that most of these relatively high exposures were caused by placing the dosimeter very close to a clock radio during the night or close to an electric appliance with a transformer. When the exposure out of the period of sleep is considered, the mean exposures for children have been found to be 0.05 µT (AM) and 0.02 µT (GM). In 11 children (1.1%), the AM values have been found stronger than 0.4 µT. The mean 50 Hz magnetic field exposures were stronger for adults than for children. The children were more exposed inside the home than outside, while the opposite was for adults. At home, both adults and children were more exposed during the day than during the night [45,46,47]. 

In 2011, a measurement campaign based on personal measurements was carried out in Italy about occupational and environmental exposure to extremely low frequency magnetic fields [48]. The objective of this study was to provide an evaluation of current magnetic field exposure of 543 workers for two days. They were monitored at work, at home and outside the home, with resulting median exposure levels of 0.14, 0.03, and 0.05 µT, respectively.

WHO summarised measurement studies in the ELF range conducted until 2007 [1]. In all studies, the majority of studied persons were exposed to magnetic field levels below 0.1 μT (73.6%–89.9%), but a few (0.5%–4.5%) had exposure levels above 0.3 μT (based on arithmetic means). Geometric means were only available for a small number of studies, but more than 90% had exposures of more than 0.1 μT and 0.4%–1.2% had exposures of more than 0.4 μT. The electric field component was more difficult to assess as it is more susceptible to shielding and perturbation by conduction bodies. 

A health impact assessment on childhood leukaemia potentially attributable to ELF-MF exposures of the general public in Europe presented pooled distributions of exposure based on a systematic review of peer-reviewed literature [49]. Exposure to ELF-MF of the general population of Europe was thus estimated as a log normal distribution with a median of 0.02 µT and a standard deviation of 0.06 µT; 0.54% of the population was considered as having an exposure of ≥0.3 µT. Importantly, this study highlighted that many existing exposure assessments and epidemiological studies have concentrated on estimating or measuring exposure among those specific subgroups of the population with stronger than average exposures, and the proportion of the general population in Europe represented by such groups is unclear.

The European Commission Scientific Committee on Emerging and Newly Identified Health Risks (SCENIHR), summarizing individual exposure levels stated that only a few percent of the European population are exposed to levels above a median magnetic field of 0.2 μT in their homes [50]. 

### 2.3. Exposure Characterization of EMF Emitting Devices 

For the general public, the highest ELF-MF are found in close proximity to household and other consumer electric appliances, and these fields may reach up to a few mT. However, these high fields are very localized and are limited to very short distances (less than some centimetres) from the surface of the equipment. According to SCENIHR, the maximum possible exposure next to a specific source often differs by some orders of magnitude from the average for individual exposure [50]. For assessment of compliance with exposure limits, the maximum possible exposure next to devices must be measured. Additionally, the exposure times are usually also limited for short-term use. The highest exposures in the ELF-MF range occur during the use of electric appliances that are held in close proximity to the body, as for example, the use of electric razors or hair dryers [1].

In a survey of 50 homes in the UK, magnetic fields were assessed at various distances from domestic appliances. The 50 Hz fundamental and harmonic magnetic fields generated by 806 domestic appliances found in the houses were measured. Appliances were measured at standard distances and magnetic fields were calculated from a mathematical model at 100 and 50 cm to remove room background contributions [51]. The fields generated by a few appliances were in excess of 0.2 μT at a distance of 1 m: microwave cookers produced 0.37 μT; washing machines 0.27 μT; dishwashers 0.23 µT; some electric showers 0.11 µT; and can openers produced 0.2 µT. Of continuously operating devices, only three devices produced fields of some tens of μT at 0.5 m: central heating pumps produced 0.51 μT, central heating boilers 0.27 μT and fish-tank air pumps produced 0.32 μT. In any case, one should note that persons spend on average about 4.5 h per day in the kitchen, where the strongest sources of magnetic fields were located [51].

In a German study, exposure to the magnetic fields from household appliances was quantified as net appliance-years of lifetime use and cumulative µT-hours, on the basis of measurements of appliances available in the published literature. Exposure was assessed on three different levels of precision: ever use, cumulative appliance-years, and average time of daily use [52]. Altogether, use of 8454 appliances was reported in the structured interview of the 3041 subjects. In total, 152,580 appliance-years were reported of which TVs had the greatest share (107,704 years), followed by microwave ovens (16,134 years), and electric blankets (10,375 years). The lifelong cumulated values were calculated from the questionnaire information on average time of daily use, the number of appliance-years, and estimated average magnetic field of the appliances. Electric alarm clocks and electromechanical digital alarm clocks had the highest number of µT-hours of all appliances (7,688,126 and 2,854,417 µT-hours, respectively) followed by electric blankets (1,695,462 µT-hours) and TVs (498,152 µT-hours). Moreover, these data suggest that one third of the total exposure to magnetic fields can be attributed to personal appliance use. Considering an average exposure of 12 h per day, a 10-year cumulative exposure would, therefore, add up to 1752 µT-hours from outdoor sources in residences. Another example is the daily exposure of workers with an 8 h working day and a 5-day working week, where cumulative exposure in 10 years would be roughly 6250 µT-hours [53]. These exposures are still well below the exposure caused by, for example, use of an electric alarm clock next to the bed assuming that a 10-year cumulative exposure would total 21,608 µT-hours. 

More than 1000 electric appliances have been investigated in Austria regarding their emission of magnetic fields. It was found that complex frequency spectra measured from 5 Hz to 2 kHz are common and single frequency (i.e., 50 Hz) emissions are rare. Usually, the devices emit complex frequency spectra, particularly those with electronically switched power control and/or electric motors.

Starting in 2009, certain types of incandescent light bulbs are being withdrawn from the market in EU and elsewhere. However, compact fluorescent lamps that are among the candidates to replace them produce IF electric fields much stronger than any other device or appliance previously available to the general public. Energy saving lamps produce fields at about 30–60 kHz. The frequencies vary slightly between different types of lamps. As with other electric appliances, they also generate ELF fields [54,55]. Results of measurements of the electric fields showed that the maximum electric field strength in the 1.2–100 kHz frequency range in close proximity to the lamps was higher than 42 V/m for all tested lamps. In nine cases out of 17, the field strength exceeded 87 V/m, and the highest measured value was 216 V/m [38]. The estimated induced current density of all investigated bulbs at a separation of 20 mm were within the ICNIRP guidelines, mostly with large margins. However, based on the large observed variations between the bulbs, it cannot be concluded that energy saving bulbs are intrinsically compliant with the ICNIRP guidelines [10].

The above-described characterization of the ELF-EMFs exposure allows classifying the exposure of European population to ELF-EMFs, so providing a useful tool to be used in the “exposure assessment phase” (i.e., the estimation of the actual dose of ELF-EMFs to which the population is exposed) of health risk assessment processes. Moreover, it is a matter of fact that a good classification of the exposure is a condition sine qua non to achieve an effective and reliable estimation of the potential health impact of exposure to any physical agent.

Table 2 shows the proposed classification of ELF-EMFs derived directly from the processing of the data detailed above. Three main classes have been identified: (i) intermittent variable partial body exposure; (ii) continuous elevated level whole body (WB) exposure and (iii) continuous low level background exposure. In summary, the first class is typically characterized by non-spatially uniform magnetic fields, decreasing quickly with the distance from the source itself, i.e., due to sources relatively close to the exposed subject. The second and the third classes are both related to far field exposure, i.e. spatially uniform magnetic field. These types of sources are typically located far away from the exposed subject. 

## 3. Conclusions

In general terms, the average exposure to low frequency magnetic fields of the general public in European countries is very low, between 0.01 and 0.1 µT. Approximately 0.5% of the general public is exposed for longer periods to levels above 0.2 µT from the fixed outdoor ELF-EMFs sources (i.e., high-voltage power lines, lines of transport systems). Elevated ELF exposure (up to a few µT) has been measured in apartments very close to built-in power transformers. The major part of exposure to magnetic fields originates from household electric devices that are used commonly by the general public but the duration of such exposure is extremely limited. In terms of cumulative exposure, approximately one third of the total exposure experienced by an individual can be attributed to the use of personal appliances. One of the exceptions is electric underfloor heating, which can lead to the exposure of all inhabitants of a house both day and night. 

In conclusion, the ELF-MF levels to which the European population is exposed are, in general terms, significantly below both exposure limits in Europe and the 0.3–0.4 µT level used to cluster the exposure data for epidemiological purposes. 

Although the data provided by this study confirm that the European population is exposed to an extremely low level of magnetic fields, well below the maximum exposure levels suggested by the international ICNIRP guidelines in 2010 [10] and in 1998 [9] which proposed limits at 50 Hz up to 200 and 100 µT, respectively, they are crucial for any future health risk assessment procedure focused on ELF-EMFs exposure, in which a comprehensive estimation of the levels of exposure (i.e., of the dose) is a fundamental prerequisite. 

One should also note that most of the reports and publications about monitoring fundamental technical information (such as the data about uncertainties of the measurements and calibration of the measurement devices) are only partially available. 

Moreover, new technological developments in Europe during recent years are leading to the introduction of new sources of low frequency fields. For instance, LED lamps are being used more and more instead of energy-saving lamps, inductive battery charging for electric cars (not only hybrids) are bearing exposure potential for occupants staying nearby, and others present some future challenges for the exposure of the general population. These new sources are not considered in this study because no sufficiently systematic data are yet available about the related exposure. However, it is easy to expect that, in the very near future, ad-hoc measurement campaigns will provide the needed data, allowing to gain a complete picture of the exposure levels at those frequencies with the inclusion of the contribution of these new sources.

## Figures and Tables

**Table 1:** Low frequency EMFs public exposure assessments in 15 European countries. There are several European countries for which we could not identify any measurements.

Country	Method of Exposure Assessment		
	Survey by in-Situ Measurements	Personal Exposimetry	Exposure Modelling
Austria	√	√	√
Bulgaria	√	-	-
Cyprus	√	-	-
France	√	√	-
Germany	√	√	√
Greece	√	-	-
Hungary	√	√	-
Ireland	√	-	-
Italy	√	√	√
Netherlands	√	√	√
Slovenia	√	-	√
Spain	√	-	-
Sweden	√	-	√
Switzerland	√	-	√
UK	√	-	√

“√ “Available; ”-“ Not Available.

**Table 2:** Classification of public exposures to ELF-EMFs.

Exposure Classification	Description, Main Sources and Relevance for Risk Assessment
**Intermittent variable partial body exposure**	- Highest level of exposure category, including the exposures from household electric devices. The exposure levels are highly variable and partial body or local. The exposure is intermittent and limited in time. The levels of exposure are below the recommended European exposure limits but the local maximum may be close to, and in some cases could be stronger than the EU reference levels reaching up to few hundreds of µT. - Typical sources: household appliances, some transport systems. - This category is considered in the risk assessment studies of general public exposure to ELF-MF that have been performed so far.
**Continuous elevated level whole body exposure**	- Medium level of exposure category including exposures from built-in transformers, and high voltage power lines within 20–50 m. The exposure levels are variable in time and space. The mean and maximum exposure levels are well below the recommended European exposure limits, reaching up to few µT. - Typical sources: built-in transformers, substations, and high voltage power lines. - This category has importance for risk analysis, when epidemiological studies on childhood leukemia are considered.
**Continuous low level background exposure**	- Low level of exposure category as the background of todays electromagnetic environment. The exposure is continuous. The mean and maximum exposure levels are many times below the recommended European exposure limits and are in the range of 0.01–0.1 µT. - Typical sources: low-voltage wiring systems, continuously operating household electric devices. - This category has very limited importance for risk analysis.
